# Practical Design Considerations for Performance and Robustness in the Face of Uncertain Flexible Dynamics in Space Manipulators

**DOI:** 10.3389/frobt.2021.708388

**Published:** 2021-09-17

**Authors:** Connor Holmes

**Affiliations:** Guidance Navigation and Control, MacDonald Dettwiler and Associates, Brampton, ON, Canada

**Keywords:** robust control, space manipulators, flexible link modes, uncertain parameters, force control

## Abstract

Low frequency dynamics introduced by structural flexibility can result in considerable performance degradation and even instability in on-orbit, robotic manipulators. Although there is a wealth of literature that addresses this problem, the author has found that many advanced solutions are often precluded by practical considerations. On the other hand, classical, robust control methods are tractable for these systems if the design problem is properly constrained. This paper investigates a pragmatic engineering approach that evaluates the system’s stability margins in the face of uncertain, flexible perturbation dynamics with frequencies that lie close to or within the bandwidth of the nominal closed-loop system. The robustness of classical control strategies is studied in the context of both collocated (joint rate) and non-collocated (force/torque and vision-based) feedback. It is shown that robust stability and performance depend on the open-loop control bandwidth of the nominal control law (as designed for a simplified, rigid plant). Namely, the designed bandwidth must be constrained to be lower than the minimum flexible mode frequency of the unmodeled dynamics by a given factor. This strategy gives credence to popular heuristic methods commonly used to reduce the effect of unmodeled dynamics in complex manipulator systems.

## 1 Introduction

Historically, manipulators designed for on-orbit operations exhibit considerably lower frequency flexible modes than their earthbound counterparts. On Earth, as the designed workspace and payload of a manipulator increases, it is common for the manipulator design to increase in rigidity and, therefore, overall mass. This reduces inaccuracies due to gravity load “sag” and moves the lowest natural frequencies of the manipulator links outside of the control bandwidth. Moreover, the workspaces of industrial, Earth-based manipulators are typically below 5 (m). Although the use of transmissions with significant compliance (such as Harmonic Drives) has become common-place in Earth manipulators, a considerable amount of the manipulator control literature has been dedicated to dealing with this problem. Most solutions involve modifying joint sensor design to include either output torque sensing or output position sensing to ensure robustness and maintain control bandwidth despite transmission compliance (Ghorbel et al., 1989; Albu-Schäffer et al., 2007; Brogliato et al., 1995).

In contrast, on-orbit manipulators do not need to contend with gravity loading, but are constrained by the need to reduce mass, thereby reducing launch costs. Additionally, such manipulators typically require larger workspaces [5 (m) to 15 (m) (4)] to be able perform large-scale assembly tasks. The combination of low mass and long links results in increased slenderness. To exacerbate this issue, the payloads that on-orbit manipulators are required to handle are considerably more massive than those dealt with on Earth, exceeding 100,000 (Kg) in the case of the Space Station Robotic Manipulator System (SSRMS) design (Laryssa et al., 2002). The increased slenderness and payload mass contribute significantly to the decrease of minimum natural frequencies of space manipulators, which are often less than 1 (Hz). As with Earth-based manipulators, joint flexibility can also contribute to the decrease of natural frequencies.

While the control of manipulators with high link compliance has been addressed in the controls literature, it has been found that many of the solutions presented in the literature are prohibitive in practice. Firstly, mode suppression solutions (as in (Luo, 1993; Sabatini et al., 2012)) that rely on sensors along manipulator links to determine vibrational mode amplitude, frequency, or shape are untenable due to the number of additional sensors required. Space qualified sensors are considerably more expensive than non-qualified sensors (by an order of magnitude in many cases), leading to significant increase in the overall cost of the system. Moreover, the introduction of additional sensors has considerable impact on avionics architecture as well as cable management, problems that can often be overlooked in the design of robotic systems.

Over the past decade, there has been a considerable shift in the space manipulator industry from large government programs to smaller commercial programs. Unlike some government programs, the budget of commercial space robotics programs cannot support a full analysis of flexible manipulator mode shapes and often must resort to simplifying assumptions. Although the resulting analyses can provide bounds on minimum flexible frequencies that advise control design, it cannot furnish advanced control syntheses that depend on detailed a priori knowledge of flexible mode shapes as in (De Luca et al., 1998). Even with such information, the author has found that the current state of the art of “space-rated” computers and microcontrollers cannot support online computation of flexible modes (Bayo, 1987; Bayo et al., 1989) or the control strategies that rely on exact, full-state, mode information (di Castri and Messina, 2012). Some stability guarantees can be made when actuators and sensors are collocated (Benhabib et al., 1981) (in some cases, when non-collocated (Damaren, 1999), but no guarantee on performance is forthcoming and collocation is not always viable. In industry, it is important to maintain simple performance and robustness metrics (such as gain margin, phase margin, overshoot, etc.) in order to serve the broader context of systems engineering. Even more crucially, some conclusions regarding control architecture and performance must be made even before the mechanical system is fully designed.

In this paper, the complicated dynamics of a flexible-link, multi-degree-of-freedom manipulator are condensed into a set of mass-spring-damper models in order to analyze control loop interactions in a simplified framework. In Section 2, a perturbed plant transfer function is derived, which consists of a single pole transfer function (representing the nominal, rigid plant) that is interconnected with a variable-frequency, resonant pole (representing the flexible-link plant perturbation). We assume that there is a known lower bound on the variation of this resonant frequency, as is typical in the aerospace industry. A state-space model is derived, but a classical robust control technique is also applied, which the author has found to be the most amenable to the systems level understanding that is requisite for successful manipulator design.

With the plant model in hand, a controller is defined in Section 3, which satisfies some standard robust design metrics for the nominal (rigid) system. In the context of the full manipulator system, this controller is analogous to the manipulator joint control system. The effect of the perturbation on control stability margins is characterized and a constraint on open-loop control bandwidth is derived to ensure that the nominal stability margins are maintained.

In Sections 4 and 5, representative models of vision-based servoing control and manipulator force/torque control are derived. These models augment the inner, joint control loop with outer feedback loops on the non-collocated, perturbed plant states. Stability margins and criteria are assessed for each control loop in terms of the effect of the flexible perturbation.

## 2 Single Joint Plant Model

As mentioned, it is the goal of this article to illustrate the effects of uncertain flexible link modes on the performance and robustness of space manipulator control systems. We seek to first reduce the manipulator dynamics to simplified, independent joint dynamics. Such simplifications are often necessary in industry to facilitate design choices at the systems engineering level and are thoroughly verified once a the manipulator system is fully designed.

The standard second-order equations of motion of rigid joint and link manipulators take the form shown in Eq. 1. These equations have been well studied and can be found in any robotics textbook (see (Sciavicco and Siciliano, 2012; Spong et al., 2005)).

$$M\left(q\right)\ddot{q}+C\left(q,\dot{q}\right)\dot{q}+B\dot{q}+g\left(q\right)=u+J^{T}F_{tip}$$ (1)

State variables *q*, $\dot{q}$, and $\ddot{q}$ are vectors of manipulator joint angles and their derivatives, *M*(*q*) is the configuration dependent mass matrix, $C\left(q,\dot{q}\right)\dot{q}$ represents the nonlinear Coriolis forces, *B* is a diagonal matrix represent viscous friction of the joints, *g*(*q*) is torque due to gravity load, *J* is the robotic Jacobian matrix, *F*_*tip*_ is a force/moment vector applied to the tip of the manipulator and *u* is a vector of control torque inputs each of the joints. We now make a series of simplifying assumptions:

• A1 The Coriolis terms ($C\left(q,\dot{q}\right)\dot{q}$) are negligible with respect to other dynamics terms. This is often the case in space manipulators where commanded joint rates are low (0–5 [deg/s]).
• A2 Gravity loading is negligible with respect to other dynamics terms. This assumption is common for on-orbit manipulators.
• A3 Joint gearboxes have large gear reduction ratios.
• A4 Each joint has the same joint rate controller with identical control parameters.

With these assumptions in mind, we note that the dominant dynamics of the joints can be considered uncoupled (see Chapter 6.2 of (Spong et al., 2005)). In this analysis, the approach of independent joint rate control is adopted and residual coupling torques between joints are lumped into disturbance input *d*. The linearized equation of motion of the *i*th joint is presented in Eq. 2 as a single joint dynamic model.

$$M\ddot{q}+B\dot{q}=u+d+f$$ (2)

With slight abuse of notation, *q*, $\dot{q}$, and $\ddot{q}$ now represent the joint angle of a given joint and its derivatives, *M* represents the lumped inertia of the manipulator from the *i*th joint to the tip of the manipulator about the axis of the *i*th joint, *B* represents the viscous friction of the *i*th joint, *u* represents the control torque of the *i*th joint, *d* denotes the lumped coupling torques from other joints onto the *i*th joint and *f* represents the projection of tip force *F*_*tip*_ to torque on the *i*th joint. This equation represents the “unperturbed” or “nominal” single joint model that will be the focus of this study. Hereafter, we neglect the explicit effect of input *d* in the analysis.

**FIGURE 1:**
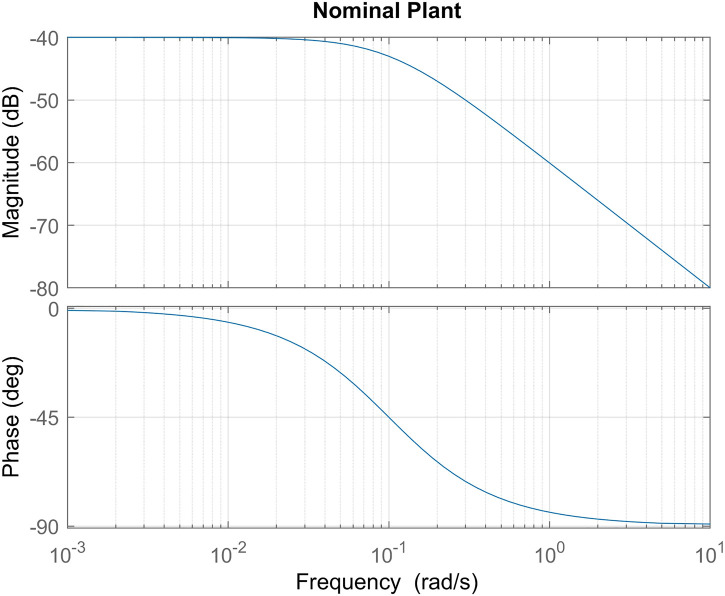
Nominal plant frequency response for joint inertia *M* =1000[*Kgm* ^2^] and viscous friction $B=100\;\left[\frac{Nm*s}{rad}\right]$

The nominal plant transfer function $P_{nom}\left(s\right):u\mapsto \dot{q}$, representing the single joint model, is given by,

$$P_{nom}\left(s\right)=\frac{1}{B}\frac{\omega_{p}}{s+\omega_{p}},$$ (3)

where *s* is the Laplace variable and $\omega_{p}=\frac{B}{M}$ is the nominal plant pole. *P*_*nom*_(*s*) represents the nominal, free-space plant transfer function from inputs *u* or *f* to joint rate $\dot{q}$ when the other inputs are ignored. For this analysis, we also make the assumption that the nominal plant pole is much lower than the desired control bandwidth *ω*_*BW*_ (i.e. *ω*_*p*_ ≪ *ω*_*BW*_). This assumption corresponds to cases in which the joint inertia *M* is large. Cases of large joint inertia are of greatest concern for this analysis, as they coincide with low-frequency, flexible dynamics. The frequency response of the nominal plant dynamics are shown in Figure 1 below.

### 2.1 Flexible Link Perturbation

**FIGURE 2:**
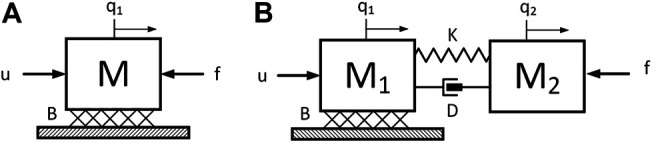
Free body diagrams of the simplified nominal model **(A)** and the perturbed model **(B)**.

Implicit in the above derivation is the assumption that the robotic links are rigid. We now consider the effect of relaxing this assumption. In general, the introduction of link flexibility into a robotic system model leads to a set of infinite dimensional, nonlinear equations of motion which considerably complicate the task of design for the control system engineer (see, for example (Macchelli et al., 2007)). Conversely, it is a goal of this paper to first explore the effects of flexible dynamics on a simplified model, thereby shedding light on the engineer’s understanding of the nonlinear, flexible robotic model. Thus, to maintain the simplicity of the model in Eq. 2, we exploit linearity and orthogonality of structural modes to model the effect flexible link modes with a single flexible perturbation of variable frequency. Free body diagrams of the nominal and perturbed models are shown in Figure 2.

The parameter *M* has been subdivided into two separate inertias connected by a spring-damper. This forms a “lumped parameter” vibrational mode perturbation, with natural frequency and damping depending on parameters *K* and damping *D*. The system of equations that represent the perturbed model are as follows.

$$\begin{array}{ll} M_{1}{\ddot{q}}_{1} & =-B{\dot{q}}_{1}-f_{p}+u, \end{array}$$ (4)

$$\begin{array}{ll} M_{2}{\ddot{q}}_{2} & =f_{p}-f, \end{array}$$ (5)

$$\begin{array}{ll} f_{p} & =K\left(q_{1}-q_{2}\right)+D\left({\dot{q}}_{1}-{\dot{q}}_{2}\right), \end{array}$$ (6)

where *q*_1_ and *q*_2_ represent the collocated and non-collocated states of the perturbed model, respectively. In the context of a full manipulator model, *q*_1_ can be interpreted as the sensed output position of the joint while *q*_2_ represents the effective joint position when link flexibility is taken into consideration. By extension, *M* is divided into *M*_1_, the inertia that is rigidly connected to the joint output, and *M*_2_, the inertia that is free to vibrate (i.e. *M* = *M*_1_ + *M*_2_). Accordingly, the viscous friction *B* generates a torque that applies only to *q*_1_. Torque *f*_*p*_ represents the coupling torque between states *q*_1_ and *q*_2_ and is determined by stiffness and damping parameters *K* and *D*. It is important to note that in this broader context of the manipulator, the state *q*_2_ is generally unobservable and is therefore not available for direct feedback control of joint rate.

The state space model of the system given by Eqs 4–6.

$$\left[\begin{array}{c} {\dot{q}}_{1}\\ {\ddot{q}}_{1}\\ {\dot{q}}_{2}\\ {\ddot{q}}_{2} \end{array}\right]=\left[\begin{array}{cccc} 0 & 1 & 0 & 0\\ -\frac{\alpha }{1-\alpha }\omega_{o}^{2} & -\frac{\alpha }{1-\alpha }2\xi \omega_{o}-\frac{1}{1-\alpha }\omega_{p} & \frac{\alpha }{1-\alpha }\omega_{o}^{2} & \frac{\alpha }{1-\alpha }2\xi \omega_{o}\\ 0 & 0 & 0 & 1\\ \omega_{o}^{2} & 2\xi \omega_{o} & -\omega_{o}^{2} & -2\xi \omega_{o} \end{array}\right]\left[\begin{array}{c} q_{1}\\ {\dot{q}}_{1}\\ q_{2}\\ {\dot{q}}_{2} \end{array}\right]+\left[\begin{array}{cc} 0 & 0\\ -\frac{1}{1-\alpha }\frac{1}{M} & 0\\ 0 & 0\\ 0 & -\frac{1}{\alpha }\frac{1}{M} \end{array}\right]\left[\begin{array}{c} u\\ f \end{array}\right],$$ (7)

where $\alpha =\frac{M_{2}}{M}$ parameterizes of the fraction of the nominal inertia that is free to vibrate, $\omega_{o}=\sqrt{K/M_{2}}$ parameterizes the natural frequency of vibration, and $\xi =\frac{D}{2M_{2}\omega_{o}}$ parameterizes the amount of structural damping in the vibrating inertia (modeled as viscous damping).

### 2.2 Perturbation in Free Space Motion

Although the state space formulation provides a complete picture of the dynamics, it does not convey an easily accessible understanding of the effect of the perturbation on the nominal dynamics of the system. To gain further insight, the dynamics are reformulated using the robust control approach to separate the nominal plant and the perturbation (see (Gu, 2013)).

Under the assumption that the joint is moving in free-space, it can be assumed that *f* = 0. Eqs. 5 and 6 can be used to find a relationship between the states of the system:

$$T_{21}=\frac{q_{2}}{q_{1}}=\frac{2\xi \omega_{o}s+\omega_{o}^{2}}{s^{2}+2\xi \omega_{o}s+\omega_{o}^{2}}$$ (8)

Summing Eqs 4, 5 and substituting Eq. 8, we see that the perturbed plant transfer function $P\left(s\right)=\frac{{\dot{q}}_{1}}{u}$ can be written as the nominal plant multiplied by a perturbation function Δ(*s*).

$$\begin{array}{l} P\left(s\right)=P_{nom}\left(s\right)\Delta \left(s\right) \end{array}$$ (9)

$$\begin{array}{l} \Delta \left(s\right)=\frac{1}{1-P_{nom}\left(s\right)\Delta_{1}\left(s\right)} \end{array}$$ (10)

$$\begin{array}{l} \Delta_{1}\left(s\right)=\frac{M_{2}\;s^{3}}{s^{2}+2\xi \omega_{o}s+\omega_{o}^{2}} \end{array}$$ (11)

**FIGURE 3:**
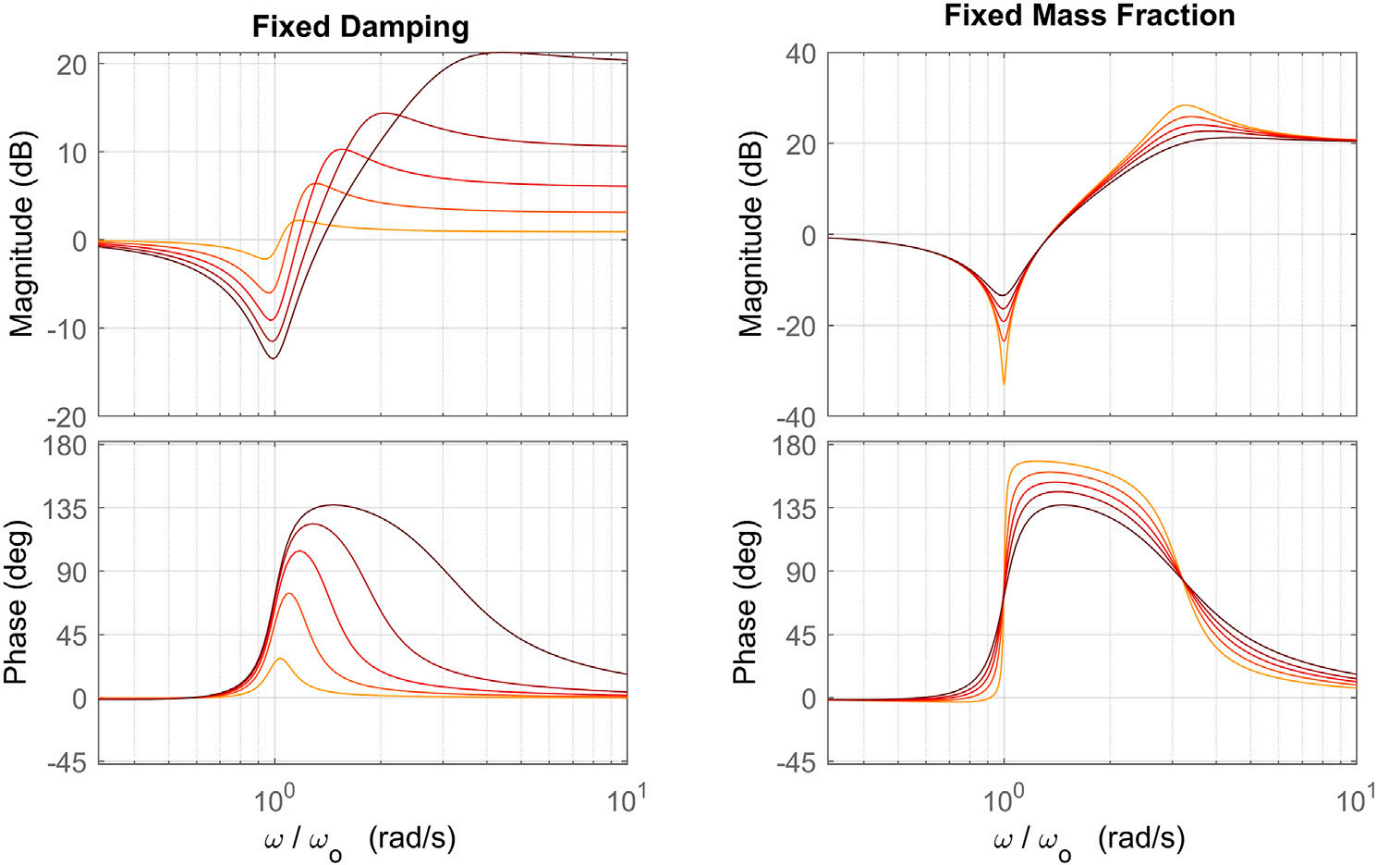
Frequency response of the perturbation with frequency normalized to the frequency parameter *ω*_*o*_. The plot on the left shows variation of the response with inertia fraction *α* ∈ {0.1,0.3,0.5,0.7,0.9} (darker color indicates higher *α*) and fixed damping *ξ* = 0.1, while the plot on the right shows variation of the perturbation with damping *ξ* ∈ {0.01,0.03,0.05,0.07,0.1} (darker color indicates higher *ξ*) and fixed inertia fraction *α* =0.9.

The frequency response of Δ(*s*) shown in Figure 3 can lend insight into the effect of the perturbation on the nominal plant. The perturbation has little to no effect at frequencies below *ω*_*o*_, but increases the plant gain at frequencies above *ω*_*o*_. If we make the approximation that the nominal plant pole is far below this frequency (*ω*_*p*_ ≪ *ω*_*o*_), we obtain the following expression for the perturbation:

$$\Delta \left(s\right)≃\frac{1}{1-\alpha }\frac{s^{2}+2\xi \omega_{o}s+\omega_{o}^{2}}{s^{2}+\frac{2\xi \omega_{o}s}{1-\alpha }+\frac{\omega_{o}^{2}}{1-\alpha }}$$ (12)

**FIGURE 4:**
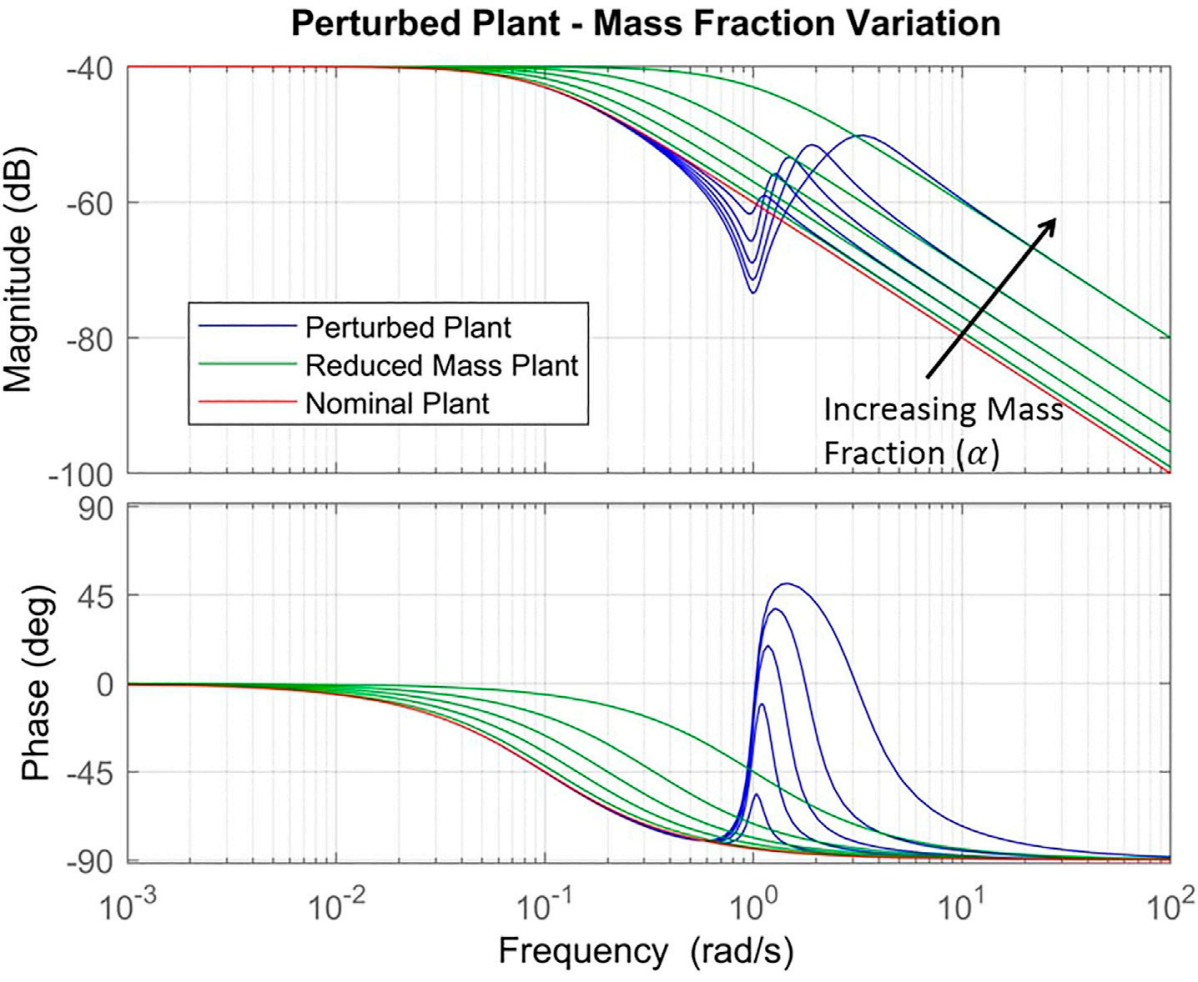
Perturbed plant transfer function is shown (blue) for *α* ∈ {0.1,0.3,0.5,0.7,0.9} and fixed damping *ξ* =0.1. The nominal plant is shown (red) as well as the nominal plant with inertia reduced to *M*_1_ (green). Black arrow indicates direction of increasing mass fraction *α*.

From this equation, we see that parameter *α* controls both high frequency gain and the width of the transition between unity and high gain regions. Figure 4 shows the nominal plant, perturbed plant and the nominal plant with inertia reduced to *M*_1_. We see from this figure that the perturbation facilitates a transitions between the nominal plant with total inertia *M* to the same plant with reduced inertia *M*_1_. This implies that the perturbation effectively separates the vibrating portion of the inertia from the joint at frequencies above $\sqrt{\frac{\omega_{o}^{2}}{1-\alpha }}$ and rigidly connects the joint inertias at frequencies below *ω*_*o*_. This insight is valuable in the consideration of control strategies for flexible link manipulators.

## 3 Joint Control Design

In this section, we develop joint control system for the nominal plant; that is, a controller constructed under the assumption that the manipulator links are completely rigid. We will then proceed to determine the effects of the perturbation dynamics derived in section 2.1 on the closed-loop joint system.

### 3.1 Nominal Joint Control Design

For this analysis, the joint control system is based on control of joint rate. The following control system performance and robustness criteria are required to be met by the nominal control system (i.e. the control system that is designed for the rigid, nominal plant given in Eq. 3). Note that these criteria will also be used when studying the effect of flexible dynamics perturbation.

• PC1 The closed loop system shall have zero steady state error for a step input command (i.e. infinite DC gain).
• PC2 The closed loop system shall have a gain margin of 10 (dB).
• PC3 The closed loop system shall have a phase margin of 45 (deg).
• PC4 The closed loop system shall have a gain rolloff of −40 $\left[\frac{deg}{decade}\right]$ to reduce noise and high frequency sensitivity.
• PC5 The closed loop system shall have a bandwidth of *ω*_*BW*_ (pass band filter frequency).
• PC6 The closed loop system should maximize disturbance rejection at frequencies below *ω*_*BW*_.

We select a control system structure that provides sufficient flexibility to address all of the above performance criteria. In particular, the structure is defined to be a Proportional-Integral (PI) controller with a first-order low pass filter. The control transfer function *C*(*s*) from rate error (${\dot{q}}_{err}={\dot{q}}_{cmd}-{\dot{q}}_{1}$) to control torque *u* and is defined as follows,

$$C\left(s\right)=\left(K_{P}+\frac{K_{I}}{s}\right)\frac{\omega_{f}}{s+\omega_{f}}=\frac{K_{P}\left(s+\omega_{z}\right)}{s}\frac{\omega_{f}}{s+\omega_{f}},$$ (13)

where *K*_*P*_ is the proportional gain, *K*_*I*_ is the integral gain, *ω*_*f*_ is the low pass filter cutoff frequency and $\omega_{z}=\frac{K_{I}}{K_{P}}$ is the PI zero. The loop gain of the nominal feedback system is defined by,

$$L_{nom}\left(s\right)=P_{nom}\left(s\right)C\left(s\right)=\frac{1}{s}\frac{K_{P}}{M}\frac{\omega_{f}}{s+\omega_{f}}\frac{s+\omega_{z}}{s+\omega_{p}}$$ (14)

The nominal closed loop joint rate transfer function (*H*_*JNT*_(*s*)) from a reference rate to joint rate is given by

$$H_{JNT}\left(s\right)=\frac{\dot{q}}{{\dot{q}}_{ref}}=\frac{L_{nom}\left(s\right)}{1+L_{nom}\left(s\right)},$$ (15)

where ${\dot{q}}_{ref}$ is a reference joint rate. We employ classical control system design techniques to select the control system parameters. The presence of the integrator ensures that closed loop system has zero steady-state error under step function input reference. Since the loop gain has relative degree 2, the high frequency phase lag is 180 (deg). Therefore, to ensure a phase margin of 45 (deg), the low pass filter cutoff frequency is set at the desired system bandwidth, *ω*_*f*_ = *ω*_*BW*_. The controller zero is set a factor of 10 below the system bandwidth to prevent any effect on phase margin while maximizing loop gain (10*ω*_*z*_ = *ω*_*BW*_). At frequencies close to *ω*_*BW*_, the effects of the plant pole (*s* + *ω*_*p*_) and controller zero (*ω*_*z*_) are negligible. The proportional gain is set such that the loop gain magnitude is one at the desired system bandwidth.

$${\left|L_{nom}\left(s\right)\right|}_{s=j\omega_{BW}}=\left|\frac{K_{P}}{Mj\omega_{BW}}\frac{\omega_{f}}{j\omega_{BW}+\omega_{f}}\right|=\left|\frac{K_{P}}{Mj\omega_{BW}}\frac{1}{j+1}\right|=\frac{K_{P}}{M*\sqrt{2}*\omega_{BW}}=1$$

$$\;\Rightarrow \;K_{P}=M*\sqrt{2}*\omega_{BW}$$ (16)

**FIGURE 5:**
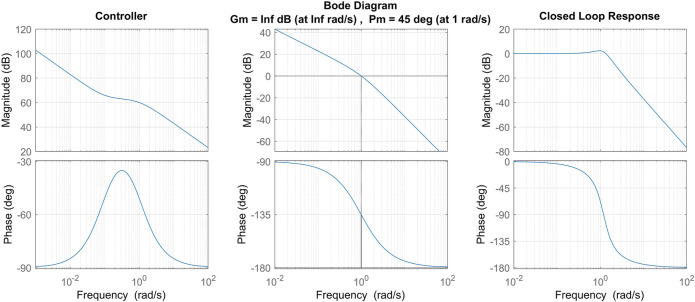
Nominal controller is shown **(left)** according to the control scheme defined in the text with *ω*_*BW*_ =1 and *ω*_*z*_ =0.1. The resulting loop gain **(centre)** is shown based on nominal controller and plant models with *M* =1000 and *B* =100. The closed loop transfer function from commanded rate to actual rate is also shown **(right)**.

Figure 5 demonstrates the designed loop gain and controller transfer functions of the design described above when *ω*_*BW*_ = 1 $\left[\frac{rad}{s}\right]$. Hereafter, it is assumed that results are normalized with respect to closed loop joint bandwidth (i.e. desired joint control bandwidth is assumed to be unity).

### 3.2 Effect of Perturbation on Control Design

**FIGURE 6:**
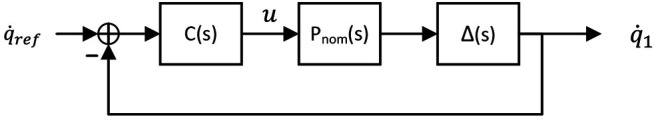
Joint control feedback loop in the presence of a vibrational perturbation.

We now explore the effect of the vibrational perturbation on the stability margins and performance of the joint control system as designed in section 3.1. Figure 6 shows the control system feedback diagram including the perturbation. The perturbed loop gain is defined as follows,

$$L\left(s\right)=P_{nom}\left(s\right)\Delta \left(s\right)C\left(s\right)=L_{nom}\left(s\right)\Delta \left(s\right)$$ (17)

Note that since Δ(*s*) always has positive phase and phase of *L*_*nom*_(*s*) never crosses Φ = −180 [deg], the gain margin of the perturbed system is infinite. This implies that the perturbation itself is not capable of causing instability when the collocated state ${\dot{q}}_{1}$ is used for feedback. Similar results regarding stability collocated feedback systems have been well studied in the literature, most notably by (Benhabib et al., 1981).

**FIGURE 7:**
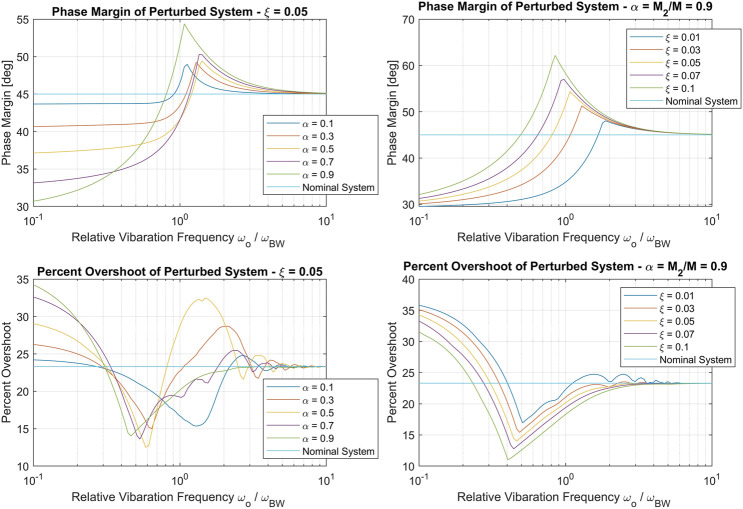
phase margin **(top row)** and Percent overshoot **(bottom row)** of the perturbed control system with vibration frequency *ω*_*o*_ normalized by designed bandwidth *ω*_*BW*_. The left column shows the effect of vibrating inertia fraction *α*, while the right column shows the effect of vibrational damping.

Regardless, the presence of the perturbation pushes the nominal control system design closer to marginal stability. Figure 7 demonstrates the effects of the perturbation on the phase margin and percent overshoot of the closed loop system. We note that as the perturbation frequency *ω*_*o*_ approaches a factor of 10 above the designed control bandwidth *ω*_*BW*_, the effect of the perturbation on robustness margin and performance becomes negligible. This lends credence to the popular industry design heuristic of bandwidth separation between control bandwidth and the minimum vibrational mode in a system. Indeed, it also reflects the fact that the perturbation function Δ(*s*) has unity gain and no phase shift at frequencies 10 times lower than *ω*_*o*_, as seen in Figure 3.

Intuitively, the margin/performance degradation is proportional to inertia fraction *α* and inversely proportional to vibrational damping *ξ*. That is, the perturbation has a more negligible effect when a smaller fraction of mass vibrates and when there is higher modal damping. It is also interesting to note that, for some parameter combinations, the flexible mode has a robustifying (margin increase) effect at relative frequencies greater than one.

## 4 Vision-Based Servoing Control

In this section, we consider the control problem of driving a manipulator to a desired pose based on a tip-mounted vision system. In its simplest form, this type of system is implemented as a proportional controller acting on tip position error. The resulting command is then processed to generate the commands for the joint-level control system. The error in vision system accuracy is neglected in this analysis. Relying on the assumption of slow manipulator rates and linearization, this problem can, again, be framed in the context of independent joint control systems.

**FIGURE 8:**
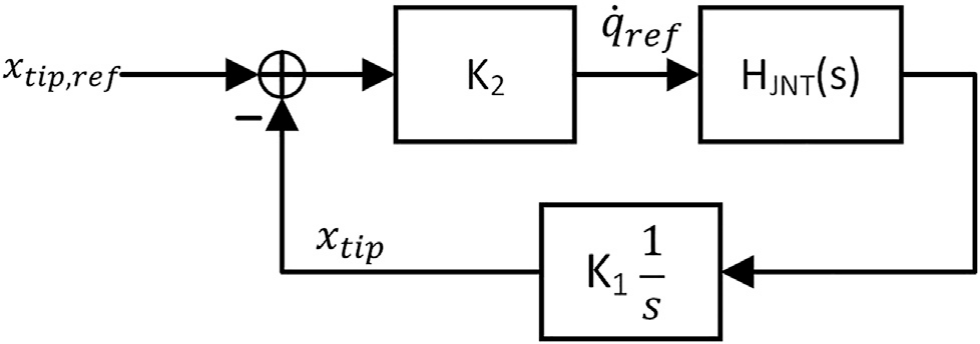
Vision-based servo loop expressed in the context of single joint control with forward kinematics lumped into *K*_1_ and inverse kinematics and tip level position gain lumped into *K*_2_.

**FIGURE 9:**
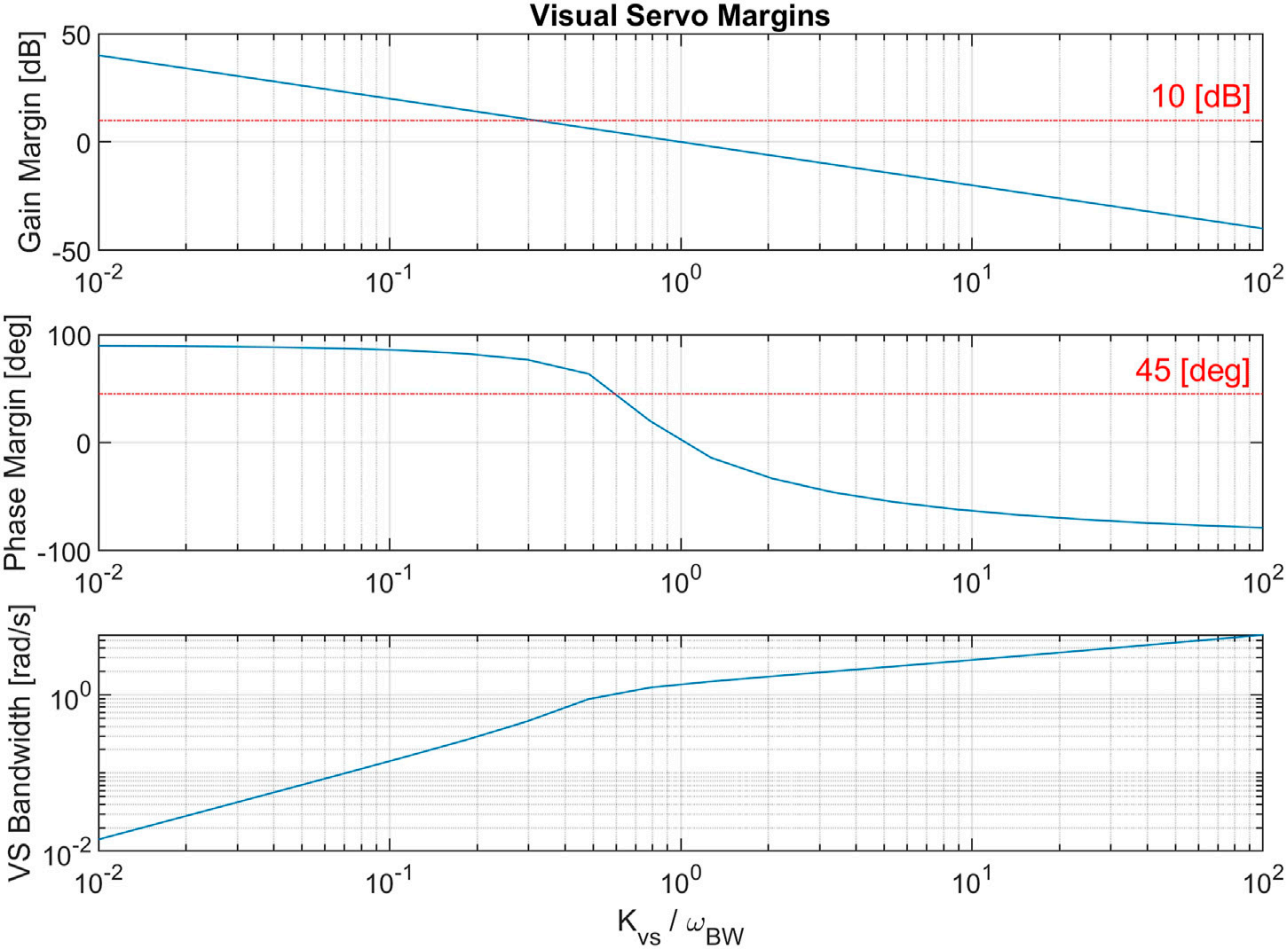
A study of vision-based servo loop margins and bandwidth based on position gain *K*_*VS*_ normalized by joint control bandwidth *ω*_*BW*_.

In such a framework, forward and inverse kinematics are lumped into fixed parameters and position feedback can be studied at the joint level. Figure 8 shows the joint level feedback loop, with *H*_*JNT*_(*s*) representing closed-loop joint rate control (see Eq. 15), *K*_1_ representing forward kinematics, and *K*_2_ representing the tip-level proportional position gain and inverse kinematics. The loop gain of the vision-based serving loop is as follows:

$$L_{VS}\left(s\right)=K_{VS}H_{JNT}\left(s\right)\frac{1}{s},$$ (18)

where *L*_*VS*_ is the loop gain and *K*_*VS*_ = *K*_1_ *K*_2_. In order to study the effects of the vibrational perturbation on this control loop, we first design the servo loop assuming that there is no perturbation. Accordingly, the closed loop joint system defined as shown in Eq. 15. Figure 9 shows robustness margins and loop bandwidth of *L*_*VS*_ as a function of gain *K*_*VS*_ normalized by joint control bandwidth. The gain is selected as *K*_*VS*_ = 0.3*ω*_*BW*_ such that *L*_*VS*_ has a 10 (dB) gain margin and 45 (deg) phase margin.

**FIGURE 10:**
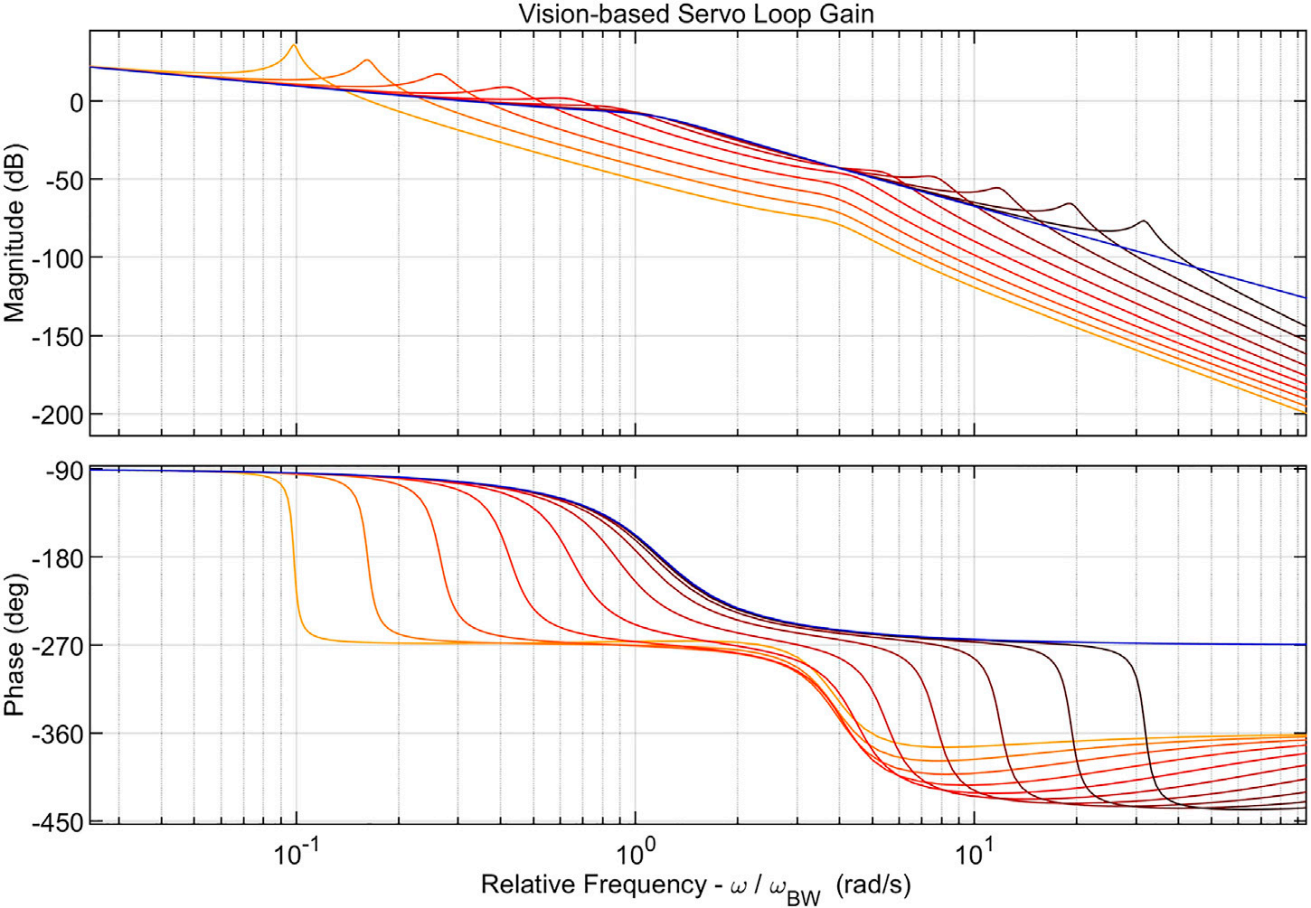
Sample frequency response plot of nominal (blue) and perturbed (red) vision-based servo loop gain. Multiple red curves correspond to the loop gain at different perturbation frequencies $\frac{\omega_{o}}{\omega_{BW}}\in \left\{0.1,10\right\}$ with darker color indicating higher frequency. Perturbation inertia fraction and damping are fixed (*α* = 0.9, *ξ* = 0.01).

We now introduce the perturbation into the joint model and study its effect on the vision-based servoing loop. Note that, with the perturbation, the closed loop joint transfer function given in Eq. 15 becomes,

$$H_{JNT}\left(s\right)=\frac{L\left(s\right)}{1+L\left(s\right)}T_{21},$$ (19)

where *L*(*s*) is the perturbed joint control loop gain and *T*_21_ (defined in section 2.1) is introduced because manipulator tip position is based on *q*_2_, freely vibrating state. A frequency response plot of the nominal loop gain compared to the perturbed loop gain is shown in Figure 10. Note that the loop gain is clearly unstable for cases where $\frac{\omega_{o}}{\omega_{BW}}<1$.

**FIGURE 11:**
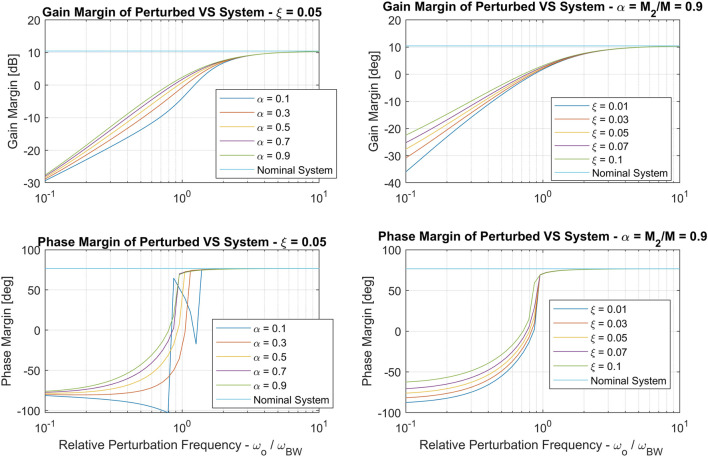
Gain margins **(top row)** and phase margins **(bottom row)** for vision-based servoing loop. The left column demonstrates the effect of changing inertia fraction *α* and the right column demonstrates the effect of perturbation damping *ξ*.

Figure 11 shows the gain and phase margins of the perturbed vision-based servoing loop gain. As expected, the stability margins are degraded whenever $\frac{\omega_{o}}{\omega_{BW}}<10$ and instability occurs when $\frac{\omega_{o}}{\omega_{BW}}<1$. Note that stability margins are fairly insensitive to values of *α* and *ξ*, but are very dependent on the frequency *ω*_*o*_.

## 5 Tip Force/Moment Control

In this section, we investigate active control of manipulator forces and torques, as reported by a force/torque sensor at the tip of the manipulator. In order to model this interaction, we assume that the manipulator is in constant contact with the environment and that the manipulator is in a “quasi-static” state. As in Section 4, we use these assumptions to model the control loop at the joint level, lumping kinematic transformations together with control gains and environment parameters.

**FIGURE 12:**
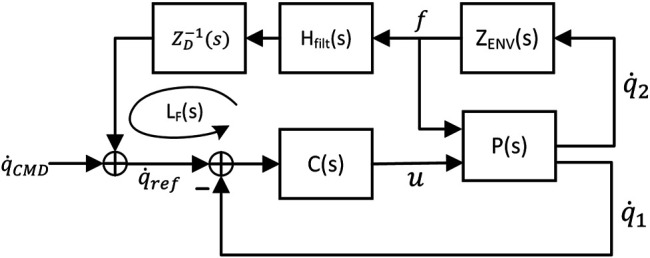
Control loop diagram for force/moment control.

**FIGURE 13:**
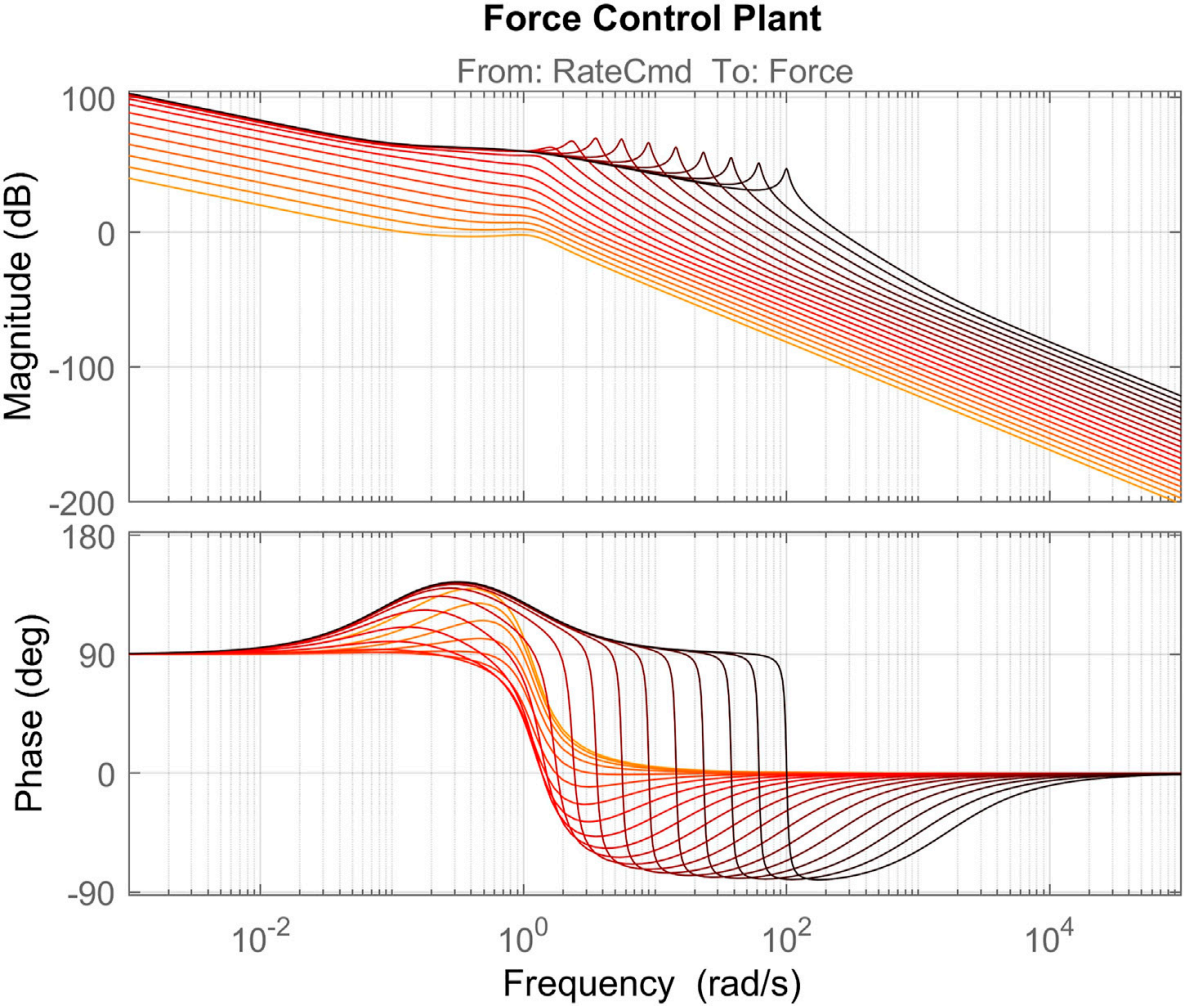
Nominal force control plant including closed loop joint control. Different lines represent the plant with different values of *ω*_*e*_ ∈ {0.01,100}, sampled evenly in log-space. Darker red color indicates higher environment natural frequency, *ω*_*e*_.

**FIGURE 14:**
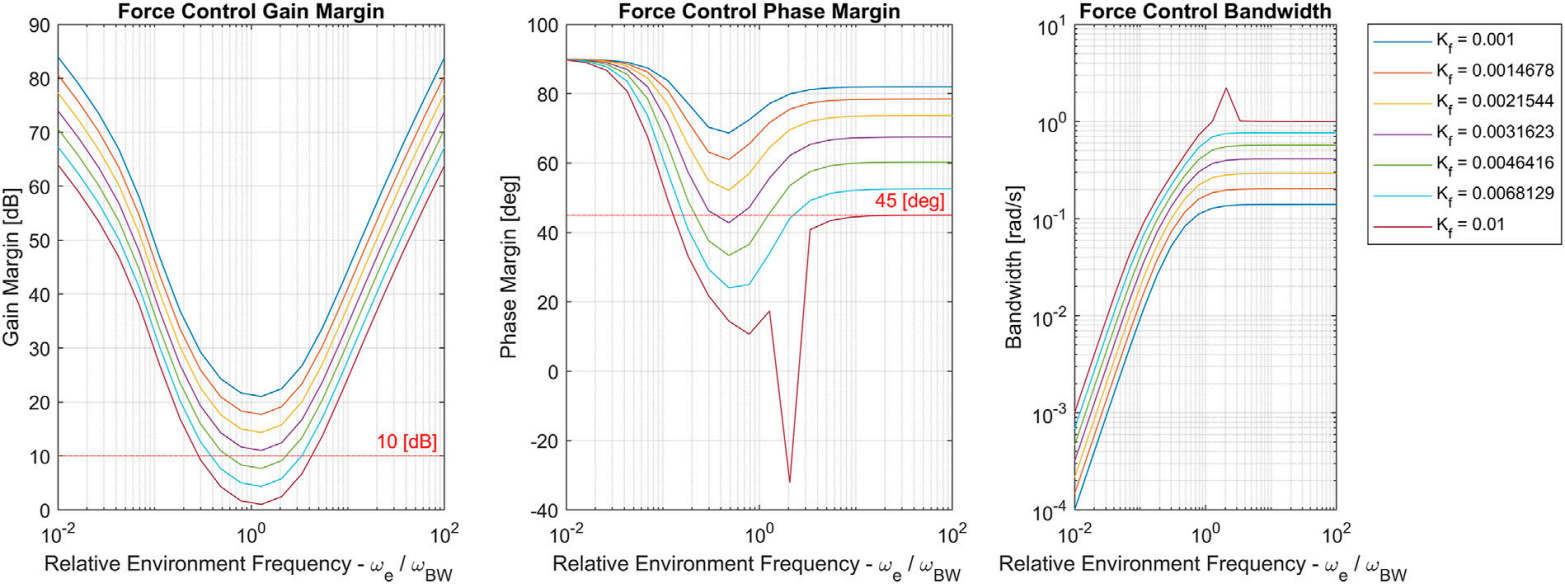
Force control gain margin, phase margin and bandwidth for the nominal system with different values of *ω*_*e*_ ∈ {0.01,100} (sampled evenly in log-space). Separate curves corresponds to different values of *K*_*f*_. Note that the discontinuity in the *K*_*f*_ =0.01 curve is due to its proximity to instability.

The joint level force/moment feedback loop model is shown in Figure 12. In this model, *p*(*s*) represents the state-space model in Eq. 7, *C*(*s*) is the joint level controller defined in Section 3.1, *Z*_*ENV*_(*s*) is the impedance function of the environment expressed at the joint (see (Kurfess, 2018) for the definition of impedance), *Z*_*D*_(*s*) is the desired impedance of the joint, and *H*_*filt*_(*s*) is a first-order, low pass filter. The loop arrow shown in Figure 12 defines the loop gain *L*_*f*_(*s*) that determines the stability of the force control system:

$$L_{f}\left(s\right)=Z_{D}^{-1}\left(s\right)H_{\mathrm{fi}lt}\left(s\right)P_{F}\left(s\right),$$ (20)

where *P*_*F*_ (*s*) is the force control plant transfer function, i.e. the mapping from joint control reference rate ${\dot{q}}_{ref}$ to force/torque variable *f*, including closed loop joint control. The environmental impedance is assumed to be a spring-damper system,

$$Z_{ENV}\left(s\right)=\frac{f}{{\dot{q}}_{2}}=\frac{K_{e}}{s}+D_{e}=M\frac{\omega_{e}^{2}+2\xi_{e}\omega_{e}s}{s},$$ (21)

where *K*_*e*_ is the spring constant, *D*_*e*_ is the viscous damping term. In the sequel analysis, the impedance is parameterized by a variable-frequency parameter $\omega_{e}=\frac{K_{e}}{M}$ and fixed damping parameter $\xi_{e}=\frac{D_{e}}{2M\omega_{e}}=0.03$. This reflects the fact that equivalent stiffness of the on-orbit robotic environment is seldom known *a priori*. A frequency response plot of the force control plant transfer function *P*_*F*_(*s*) as *ω*_*e*_ is varied is shown in Figure 13. The purpose of *H*_*filt*_ is to limit force control bandwidth and the level of noise of the force sensor. To maintain bandwidth separation, the cutoff frequency of this filter is set to a factor of 10 below the joint control bandwidth, *ω*_*BW*_. For simplicity, the desired impedance is a damper, i.e. $Z_{D}^{-1}\left(s\right)=K_{f}$. Figure 14 shows stability margins and bandwidth of the nominal force control loop as environment stiffness/natural frequency and gain *K*_*f*_ are varied. Based on this plot, a control gain of *K*_*f*_ = 0.003 was chosen to maintain sufficient control loop margins [10 (dB) gain margin and 45 (deg) phase margin] for the nominal system.

**FIGURE 15:**
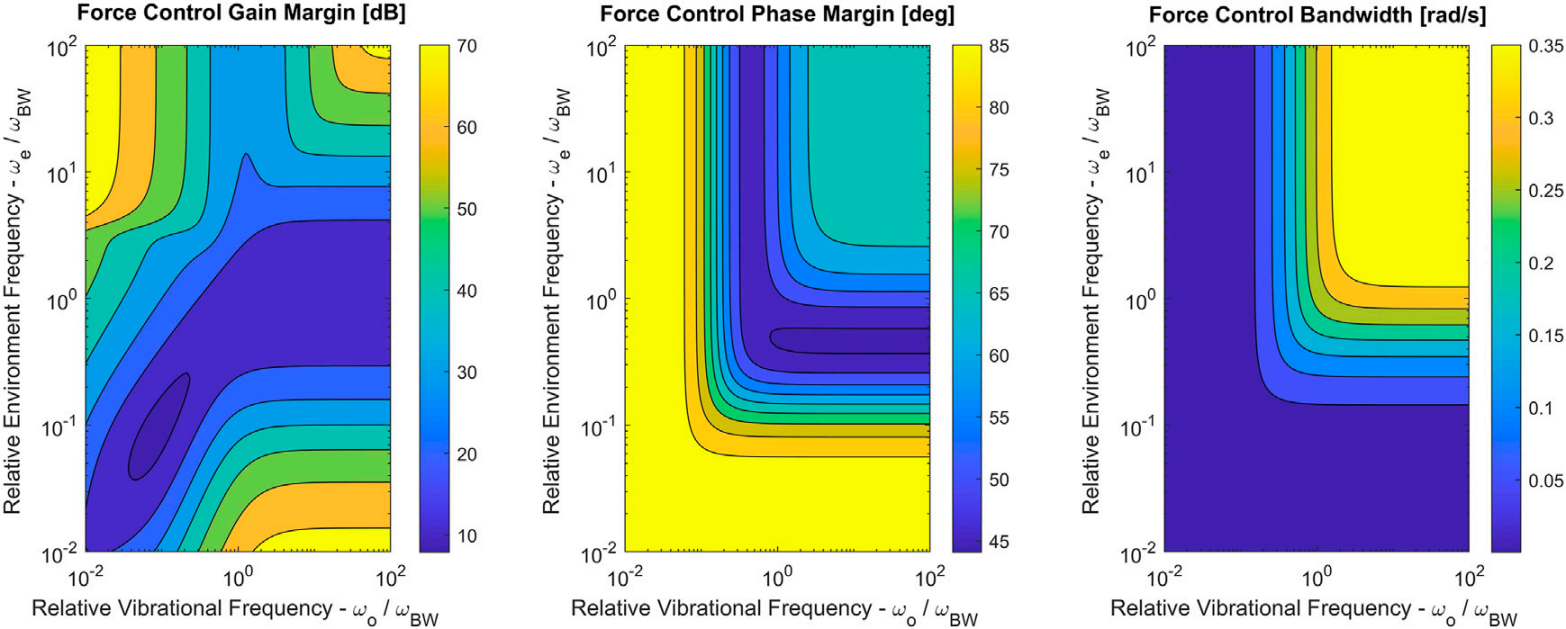
Force control gain margin, phase margin and bandwidth for the perturbed system with different values of *ω*_*e*_ ∈ {0.01,100} and *ω*_*o*_ ∈ {0.01,100} (both sampled evenly in log-space). Perturbation parameters were set to *α* = 0.9 and *ξ* = 0.04.

We now analyze the stability margins of the force control loop when the vibrational perturbation is introduced. In this case, the margins depend on both the perturbation parameter *ω*_*o*_ as well as the environment impedance frequency parameter *ω*_*e*_. Figure 15 shows stability margins and bandwidth for the perturbed force control loop.

Interestingly, there is little to no degradation of force control stability margins when the vibrational perturbation is introduced. This is likely due to the fact that the nominal system was already designed to be robust against the vibrational modes induced by the environment impedance. However, the presence of the perturbation does reduce bandwidth when environmental stiffness is high (*ω*_*e*_ > *ω*_*BW*_).

All three plots in Figure 15 suggest that as each of the frequencies increases past a threshold, it ceases to have an influence on stability and bandwidth. Intuitively, this corresponds to either the environment or the perturbation mode stiffnesses becoming near-rigid.

## 6 Conclusion

In industry, it is often necessary to develop low-order models that, despite their simplified nature, are capable of capturing physical phenomena well enough to allow sound engineering conclusions to be drawn. This is especially true when the mechanical system under analysis is not yet fully designed and, nonetheless, control decisions must be made. This is almost always the case when sufficient flexibility is introduced into on-orbit manipulators.

In this article, the complicated dynamics of on-orbit, flexible-link manipulators have been simplified into a representative single joint model with a vibrational perturbation. The nature of this perturbation was explored in the context of manipulator joint control. A condition under which stability margins and performance of the joint controller are not degraded by such a perturbation was demonstrated. This condition was found to match well with a popular heuristic for control of flexible systems, namely that designed joint control bandwidth frequency should be set a factor of 10 lower than the minimum vibrational mode frequency of the links.

Further, the effect of the perturbation was studied in the context of vision-based servoing and force/moment control at the tip of the manipulator. In the former case, the presence of the vibrational perturbation was found to result in severe margin degradation and even instability in cases where a bandwidth separation factor of 10 was not enforced. Moreover, when force/moment control is designed to be robust against a range of environment impedences (corresponding to different frequencies when coupled with the joint), additional vibrational perturbations did not degrade stability margins below their designed values. The perturbation did, however, affect the bandwidth of the force/moment control.

Though the concepts and models expounded in this article have been guided by the author’s experience with tuning and control of real (as opposed to simulated) manipulators, validation of said models is an ongoing pursuit. In terms of future work, validation of these models using data from actual hardware is foremost in the mind of the author. Such validation, however, is subject to the limitations imposed by industry research - namely, cost, data exposure, and intellectual property restrictions.

## Data Availability

The original contributions presented in the study are included in the article/Supplementary Material, further inquiries can be directed to the corresponding author.
